# A Safety Huddle Intervention in In-Patient Surgical Units: A Mixed-Methods Study

**DOI:** 10.1155/2023/8929993

**Published:** 2023-07-05

**Authors:** Seung Eun Lee, V. Susan Dahinten, Eunkyung Kim, Sang Hwa Lee, Soo Young Han, Phill Ja Kim, Jung Yeon Kim

**Affiliations:** ^1^Mo-Im KIM Nursing Research Institute, College of Nursing, Yonsei University, Seoul 03722, Republic of Korea; ^2^School of Nursing, University of British Columbia, Vancouver V6T 2B5, Canada; ^3^College of Nursing and Brain Korea 21 FOUR Project, Yonsei University, Seoul 03722, Republic of Korea; ^4^Division of Nursing, Severance Hospital, Seoul 03722, Republic of Korea

## Abstract

Open communication about patient safety concerns is necessary to enable a learning environment where lessons can be learned to improve patient safety, but nurses often hesitate to speak up even in situations where their patients may be at risk. One way to create a safe environment for speaking up is through the use of unit-level daily huddles. This study aimed to assess the effects of a 12-week huddle intervention on nine unit, nurse and patient care outcomes and describe nurses' experiences with the intervention. We used a single group, pre- and post-test mixed-methods design, with a dominant quantitative thread, and a final sample of 89 staff nurses. The intervention was conducted in four surgical units in a tertiary teaching hospital in Seoul, Korea. The intervention included two educational workshops for huddle leaders, two workshops for staff nurses, and 12-week huddles with coaching visits. We collected quantitative data on nine outcomes using online surveys before and after the intervention and qualitative data on nurse experiences of the intervention after the intervention. Paired *t*-tests were used for quantitative data analysis, and content analysis was used for qualitative data. We examined four unit-level outcomes (organizational learning, situation monitoring, mutual support, and speaking-up climate), three nurse-level outcomes (promotive and prohibitive voice behaviors and job satisfaction), and two patient care outcomes (patient safety and quality of care). Significant improvements were found in six of the nine outcomes. Findings from the qualitative data confirmed the benefits of the intervention but also identified challenges to huddle participation. Patient safety huddles can contribute to a learning environment by flattening hierarchies and encouraging nurses to speak up regarding safety issues. Leadership is a key in role modelling and creating the foundation for a more collaborative patient safety culture in healthcare organizations, for example, through the use of daily huddles.

## 1. Introduction

Although efforts have been made to enhance patient safety in healthcare over the last two decades, patients still experience preventable adverse events during hospitalization, and patient harm is the 14th leading cause of disease burden globally [1]. Open communication about patient safety concerns is necessary to enable a learning environment [2] where lessons can be learned to improve patient safety and the quality of care [3]. Among the various healthcare professionals, nurses play an important role in ensuring safe, quality care for patients. Because of their constant presence at the bedside and direct contact with the patients, nurses are usually the first to notice errors and near misses that can affect patient safety [4]; thus, it is important that they are able to voice their concerns and suggestions related to safe patient care. However, nurses often hesitate to speak up even in situations that could put their patients at risk [5].

It is widely known that speaking up is challenging for those who are at lower levels in the healthcare hierarchy, such as nurses [6]. However, in East Asian cultures, speaking up is difficult even within nursing care teams, as cultural values such as collectivism, obedience, and respect that are embedded in general society [7] are also reflected in healthcare contexts as seniority- and age-based hierarchies [5, 8]. In addition, the tendency in Korean healthcare environments to assign blame to individuals for errors [9] leads nurses to suppress their voices about patient safety concerns that involve themselves or their team members. Other identified barriers to speaking up include the lack of a safety culture, ineffective teamwork, unsupportive managers and colleagues, and the notion that no change will result from speaking up for better patient safety and quality of care [5, 10–13]. Research has shown that creating an environment where nurses feel safe to voice their concerns and suggestions without fear of punishment, retaliation, or humiliation [5, 14–16] is vital for continuous organizational learning, which will, in turn, contribute to better patient safety and quality of care [16, 17].

One way to overcome the abovementioned barriers and create a psychologically safe environment for speaking up is through the use of a unit-level daily huddle [14]. A huddle is a short, regular meeting that takes no more than 10 minutes at the start of each work day or shift in a clinical setting [18]. Regular huddles provide opportunities for team members to discuss concerns, address issues, and provide and receive feedback. Huddles provide a platform for open communication and information sharing which can flatten hierarchies in clinical settings and empower frontline staff to raise their voices [19, 20]. Huddles also help to build trust and positive work relationships, which can enhance the quality of patient care [14]. Research has shown the benefits of huddles in healthcare settings, including improved teamwork and communication [20, 21], an enhanced patient safety climate [22], and increased job satisfaction of frontline staff [21]. However, most studies have been conducted in Western cultures [23], and interventions developed in one culture do not always translate easily into other contexts [24]. To our best knowledge, no intervention research on the use of huddles has been conducted in Korean healthcare contexts. Thus, we developed, implemented, and evaluated an intervention focused on the use of huddles in surgical units in a Korean hospital.

The aim of this study was to assess the effects of the huddle intervention on nine unit, nurse and patient care outcomes. We investigated four unit-level outcomes (organizational learning, situation monitoring, mutual support, and speaking-up climate), three nurse-level outcomes (promotive and prohibitive voice behaviors, and job satisfaction), and two patient care outcomes (patient safety and quality of care), as well as nurse experiences of the intervention.

## 2. Materials and Methods

### 2.1. Study Design, Setting, and Sample

We used a single group, pre- and post-test concurrent mixed-methods design, with a dominant quantitative thread. Qualitative data were used simultaneously in data analysis at Time 2 for enriching our understanding of the quantitative findings [25]. The intervention was conducted in four surgical units (ranging from 48 to 53 beds) in a 1,099-bed, tertiary, nonprofit, teaching hospital in South Korea. Surgical units were selected as they have a higher risk of adverse events than other hospital units due to a high degree of complexity; thus, a huddle intervention might enhance patient safety in these units [26].

The principal investigator (PI) met with the chief nursing officer and two nursing directors to explain the study purpose and proposed methods. The PI then met with the unit managers of surgical wards to explain the study and ask for study participation. Using convenience sampling, four study sites were selected based on the managers' willingness to participate. Subsequently, all 108 staff nurses in the four units were invited by emails and posters in the staff room to participate in the study, and 103 consented to participate. The minimum sample size needed was 34 to reach a sufficient power (80%), effect size (0.5), and alpha (0.05), based on G-Power version 3.1.9.4. Ethical approval for this study was obtained from the Institutional Review Board of the participating university health system (#4-2021-0766). This study was conducted in accordance with the principles of the Declaration of Helsinki. Due to the nature of the online survey, no written informed consent was necessary, but participants were advised that clicking boxes regarding consent to participate on the first page of the questionnaire constituted the consent to participate in the study.

### 2.2. The Intervention

The PI, who has a graduate certificate in patient safety, developed the training materials using various resources such as the Daily Huddle Component Kit [27], the Patient Safety Essential toolkit: Huddles [18], the Safety Huddles Implementation Guide [28, 29], and the Framework for Safe, Reliable, and Effective care [30]. All materials were reviewed by two content experts.

Two 2-hour face-to-face workshops were provided to the four huddle leaders (i.e., unit managers) on leadership development, safety culture, psychological safety, and communication and teamwork with a focus on huddles as the key strategy. Two 1-hour online workshops on safety culture, psychological safety, and communication and teamwork with a focus on huddles were given to the staff nurses. Following the completion of the workshops, the research team supported the initiation of daily huddles. In each unit, the huddle took place at 7:30 am in nurse workrooms, allowing nurses to discuss concerns and issues related to patient safety in a confidential manner [31]. Each unit used a visual management board (i.e., the huddle board) for a standardized huddle. The huddle leaders were asked to document the starting and finishing time, attendance, issues addressed, concerns discussed, resolution status, and the number of patient safety issues raised during each huddle, using a huddle checklist created by the research team [18, 27, 28, 32]. Weekly coaching visits were made to each unit for the first four weeks, followed by monthly coaching visits until the intervention was completed. A huddle evaluation form developed by the research team was used when providing feedback to the huddle leaders. Also, weekly calls, when possible, were established between the PI and the huddle leaders. The intervention, including educational workshops for leaders and staff nurses and 12-week huddles, was conducted from October 2021 to February 2022.

### 2.3. Data Collection Procedures

Data were collected at the baseline (Time 1, before the initial educational workshop was held) and at the follow-up (Time 2, one week after the completion of the intervention) using an online survey. The Time 1 survey included demographic questions and measures of the nine outcomes. The Time 2 questionnaire measured the same nine outcomes but also included open-ended questions asking nurses about their experiences with the intervention.

### 2.4. Measures

Nine standardized measures were used in this study. Items for overall patient safety and quality of care were each rated on a 4-point response scale ranging from 1 (poor) to 4 (excellent). All other items were rated on a 5-point response scale ranging from 1 (strongly disagree) to 5 (strongly agree). Higher scores indicate higher levels of the construct.

Organizational learning (3 items) was assessed using the organizational learning, continuous improvement subscale from the Korean version of the hospital survey on patient safety culture (K-HSOPSC) 2.0, which has demonstrated acceptable reliability and validity in a nursing population [33]. A sample item is “In this unit, changes to improve patient safety are evaluated to see how well they worked.” Cronbach's alpha for the scale was 0.70 at Time 1 and 0.71 at Time 2.

Situational monitoring (7 items) and mutual support (7 items) were each measured using two relevant subscales from the Teamwork Perceptions Questionnaire [34], which has shown good psychometric properties [35]. Items included “Staff actively anticipate each other's need” (for situation monitoring) and “Staff assist fellow staff during high workload” (for mutual support). For the present study, the Cronbach's alpha for situational monitoring was 0.86 at both Times 1 and 2, and the alpha for mutual support was 0.81 at Time 1 and 0.85 at Time 2.

Speaking-up climate (5 items) was assessed using the speaking-up climate for patient safety, which has demonstrated good reliability and validity [36]. A sample item is “The culture in my clinical area makes it easy to speak up about a patient safety concern that does not involve me or my patients.” For the study sample, Cronbach's alpha was 0.78 at both Times 1 and 2.

Nurses' voice behaviors were measured using Liang et al. [37]'s 10-item, 2-dimensional scale for promotive voice (5 items) and prohibitive voice (5 items), which has demonstrated acceptable reliability and validity [37]. Items include “I proactively develop and make suggestions for issues that may influence the unit” (for promotive voice), and “I speak up honestly with problems that might cause serious loss to the work unit, even when/though dissenting opinions exist” (for prohibitive voice). In the study sample, the Cronbach's alpha for promotive voice was 0.93 at both Times 1 and 2, and the alpha for prohibitive voice was 0.88 at Time 1 and 0.87 at Time 2.

Nurses' job satisfaction (3 items) was measured using the job satisfaction subscale of the Michigan Organizational Assessment Questionnaire [38], which has demonstrated good psychometric properties [39]. A sample item is “All in all, I am satisfied with my job.” For the study sample, Cronbach's alpha was 0.85 at both Times 1 and 2.

Patient safety and quality of care were each measured with a single item from the K-HSOPSC [33] that asked nurses to provide an overall rating of each dimension of care in their units. Reliability has been shown for both measures (patient safety, [40]; quality of care, [41]).

Participants' background information (gender, age, educational level, years of nursing experience, hospital tenure, and unit tenure) was also asked at Time 1. At Time 2, we asked nurses to respond to the following open-ended items: (1) Please describe any changes that occurred as a result of the huddles; (2) Please describe any challenges you faced participating in huddles; and (3) Please feel free to provide any other comments about the huddles.

### 2.5. Data Analysis

All quantitative data analyses were conducted using SPSS version 25. Descriptive statistics were used to describe the characteristics of the participants and key study variables. Within-group differences in study outcomes were examined using paired *t*-tests with an alpha of 0.05. We analyzed the qualitative data using manifest content analysis [42]. Phrases and sentences were used as units of analysis. Two researchers independently reviewed the data and coded the meaning of units, repeatedly comparing their results, and discussed any differences in interpretation until an agreement was reached to assure credibility of the findings. The codes were integrated into subcategories and categories, and exemplar quotations were selected. Findings from the qualitative data were used to better understand the findings that arose from the quantitative data.

## 3. Results

### 3.1. Participant Characteristics

A total of 103 staff nurses completed the baseline survey, and 89 completed the follow-up survey. Almost all participants in the final sample were female (*n* = 86, 97%) and had a baccalaureate degree in nursing (*n* = 85, 92%). The mean age was 29.2 years (SD = 7.42) and the average length of nursing experience was 5.4 years (SD = 7.36). The mean hospital and unit tenure was 5.0 (SD = 6.3) and 2.8 years (SD = 2.4), respectively.

### 3.2. Changes in Outcome Variables

Table 1 summarizes the mean scores on each outcome variable at the baseline (Time 1) and the follow-up (Time 2). After 12 weeks of the huddle intervention, significant improvements were found in six of the nine outcomes: organizational learning, situation monitoring, nurses' promotive and prohibitive voice behaviors, and units' overall patient safety as well as quality of care. The mean scores of mutual support, speaking up climate, and nurses' job satisfaction increased but were not statistically significant.

### 3.3. Findings from the Open-Ended Items

Analysis of the qualitative data yielded 3 categories and 9 subcategories. The findings are discussed below and exemplar quotes are presented in Table 2.

#### 3.3.1. Benefits of Huddles

A majority of the participants reported that huddles promoted growth in their overall situational awareness that they were more likely to identify safety and quality issues requiring immediate or extra attention. In particular, the huddles were seen to help the less-experienced nurses develop a more comprehensive view of unit operations and procedures that can impact patient safety. Participants noted that using the visual huddle board contributed to their situational awareness. Participants also commented that having a designated time every day for communicating with their team members was critical for patient care. Sharing information about high-risk patients on the unit allowed the nurses to pay more attention to those patients, put preventive measures in place, and provide rapid responses (risk management). Nurses mentioned that identifying safety risks and potential solutions reduced patient safety incidents, including fewer patient falls. Finally, the participants reported that huddles enabled them to anticipate each other's needs which resulted in improved teamwork and collegiality. Also, creating the time and space for meaningful team communication strengthened a culture of safety and created an atmosphere that encouraged speaking up. Remarkably, two nurses reported that less experienced or newly graduated nurses felt more empowered to speak up, even with those who had more seniority and authority.

#### 3.3.2. Challenges for Huddle Participation

The most frequently mentioned challenge to huddle participation was the time pressure related to heavy workloads. Although nurses understood the importance and benefits of huddles, creating time to participate could be burdensome at times. A few nurses also mentioned that changes or a deterioration in a patient's condition meant that direct patient care took priority over participation in the huddle. Additionally, nurses noted the challenges presented by a lack of understanding by patients, caregivers, and other health professionals regarding huddles. One nurse mentioned that other healthcare providers (e.g., physicians) disturbed huddles, and it seemed that they did not understand or appreciate the importance of huddles for improving patient safety (the lack of awareness about huddles).

#### 3.3.3. Recommendations for Future Huddles

Few participants offered recommendations for future huddles. One nurse suggested the use of digital board instead of the analog huddle board. Another noted that there should be a plan for proceeding with the huddle in situations where the unit manager is unable to participate as the huddle leader. There was also a suggestion for an alarm notifying that it was time to join the huddle.

## 4. Discussion

This study examined the effects of a huddle intervention on various outcomes in surgical units in a Korean hospital. We found statistically significant improvements in six of the nine outcomes, organizational learning, situation monitoring, nurses' promotive and prohibitive voice behaviors, and overall patient safety and quality of care, following the intervention. We also gained further insights from nurses regarding their experiences with the intervention, demonstrating the benefits and challenges of huddles.

Consistent with the previous research [26], our study found that the huddle intervention improved organizational learning. Patient safety requires organizational learning, which is demonstrated by a willingness and ability to learn from feedback [43] and changes in organizational routines that impact risk and safety [44]. In the current study, sharing information between the huddle leader and staff and among team members, and the subsequent modification of patient care processes seem to have contributed to the nurses' perceptions of their ward as a learning unit for improving patient safety and quality.

Notably, we found that nurses' speaking up behaviors, an important aspect of patient safety culture [26], improved following the huddle intervention. A standardized huddle generates the necessary structure for openly discussing safety concerns. The huddle creates a common ground among team members that assists in flattening the hierarchies in healthcare teams [45]. In the current study, nurses noted that huddles created an environment that encouraged speaking up and enhanced the culture of safety. Speaking up about safety concerns in a workplace is more difficult for healthcare professionals who are at a lower hierarchical level [6] and work in an environment with poor safety culture [46] and inadequate support from their direct managers [15] and colleagues [5]. A standardized huddle intervention may be a strategy for addressing each of these issues.

Consistent with the previous research [20], we found that huddles enhanced nurses' situational awareness, for example, by discussing the potential risks of a particular medication, which would have facilitated error prevention. Although we did not have the necessary data to quantify the frequency of such events, in the qualitative responses, nurses reported that there were fewer patient safety events such as patient falls. Previous studies [47, 48] have demonstrated significant reductions in patient adverse events such as medication errors and patient falls after huddles in clinical settings. Thus, when possible, future research should investigate the effects of huddles with more objective data such as incident reports.

In the current study, we did not find a significant increase in nurse-perceived mutual support following the intervention. A prior study [26] similarly failed to find improvements in mutual support after 6 months of huddle intervention, but they did find improvements after 12 months. This could imply that mutual support should be viewed as a long-term rather than short-term outcome and suggests the importance of sustaining the huddle intervention. The intervention in our study was limited to 12 weeks for practical reasons, but the huddle has become an integral part of daily routine in the four study sites, and the hospital plans to expand the intervention to other nursing units. Management commitment and support for huddles will be the key for successful intervention implementation and sustainability [14]. In the current study, the intervention was carried out within a single discipline (i.e., nursing staff), but multidisciplinary huddles should be considered due to the reported benefits of interprofessional huddles for improving patient safety and quality of care [23]. The lack of awareness by other healthcare personnel regarding the importance of huddles that was noted as a challenge in this current study may be resolved through multidisciplinary huddles.

### 4.1. Limitations

Several study limitations should be noted. First, due to the limited number of nursing units included in this study, we were unable to account for the effects of nurses clustered within units, which may have affected the study findings. Second, we were unable to include control groups due to complications posed by the COVID-19 situation. Although this poses a threat to the internal validity of the study, a prepost design is a common and practical method in settings where a randomized design is not feasible [26]. Finally, the intervention was conducted in surgical units in one Korean hospital, and the study findings may not be generalizable to other contexts.

## 5. Conclusion

This study was the first to examine the effect of huddles among nurses in a Korean healthcare context. Our primary findings indicate that the daily safety huddle may be a useful approach for flatting hierarchies and creating an environment where frontline nurses can speak up freely about patient safety and quality of care issues and concerns. Huddles appear to improve the effectiveness of information sharing and engender a more collaborative culture that contributes to a shared awareness and anticipation of patient safety risks which will, in turn, improve patient safety and quality of care. Due to the study's limitations, there should be further evaluation of the effects of huddles with stronger research designs and broader target populations.

### 5.1. Implications for Nursing Management

Open communication and collegial support for safety concerns are vital for improving patient safety. Although all team members are responsible for creating a psychologically safe environment [49], leadership is the key in role modelling and creating the foundation for a collaborative culture in healthcare organizations. Implementing and sustaining a safety huddle is challenging in complex healthcare settings [20], and strong leadership support from managers at all levels is needed. Unit managers should encourage staff to actively engage in huddles by listening to staff concerns, considering their inputs, and providing respectful feedback, with the shared goal of increasing patient safety.

## Figures and Tables

**Table 1:** Mean scores of variables pre- and postintervention with paired *t*-test (*N* = 89).

Variables	Preintervention	Postintervention	Mean difference^a^	*t*	*p*
	*M* (SD)	*M* (SD)			
*Unit outcomes*					
Organizational learning	3.07 (0.70)	3.45 (0.61)	0.38	5.00	<0.001
Situation monitoring	3.61 (0.54)	3.73 (0.52)	0.12	2.03	0.046
Mutual support	3.60 (0.49)	3.67 (0.52)	0.08	1.31	0.195
Speaking up climate	3.44 (0.52)	3.54 (0.59)	0.09	1.62	0.110

*Nurse outcomes*					
Promotive behavior	2.55 (0.73)	2.93 (0.81)	0.38	4.60	<0.001
Prohibitive behavior	2.87 (0.72)	3.19 (0.73)	0.32	4.20	<0.001
Job satisfaction	3.15 (0.74)	3.16 (0.81)	0.01	0.16	0.876

*Patient care outcomes*					
Patient safety rating	2.36 (0.53)	2.60 (0.65)	0.24	3.08	0.003
Quality of care	2.44 (0.46)	2.82 (0.49)	0.38	6.76	<0.001

^a^Mean difference, the difference between post-test and pretest scores (post-test–pretest).

**Table 2:** The results of qualitative data analysis (*N* = 89).

Categories (*n*)	Subcategories (*n*)	Quotes
Benefits of huddles (80)	Growth in overall situational awareness (76)	(i) I was able to respond to patients without delay, as we shared important information for patient care, such as patients at high risk for falls, patients with severe delirium, and patients with multiple drainages that we would not have been aware of if they were not my patients. Huddle really helped me to understand what is going on in the unit (P 44)
		(ii) It was good and helpful to share patient safety events and potential safety risks in the unit, which I would not have known normally without huddles (P 42)
		(iii) It was helpful for newly graduate nurses to be able to get an overview of the situation in the unit (P 37)
		(iv) Through the huddle board, I was able to understand what has been going on in the unit and how the unit has been doing as a whole (P 17)
	Risk management (23)	(i) Huddles helped to prevent patient safety incidents by identifying high-risk patients (P 54)
		(ii) We can identify and pay more attention to patient safety issues that frequently occur particularly in our unit (P 39)
		(iii) Huddles helped reduce the number of patients' falls in the unit (P 22)
	Improved teamwork and collegiality (17)	(i) It was nice to help other team members when they had high workload and to be able to get help when I needed their helps (P 68)
		(ii) Huddles helped improve teamwork by identifying and supporting team members who had high workloads due to their responsibilities to take care of patients with high acuity (P 24)
	Strengthened safety culture (7)	(i) Because of huddles, an atmosphere that supports speaking up has been created; so, it seems that reporting to colleagues or to those who has more authority has become a habit, and it has been a lot earlier to speak up (P 28)
		(ii) Huddles were the beginning of creating a venue for even less-experienced nurses to express their opinions (P 63)
	Facilitated team communication (5)	(i) Efficient communication was possible because patient safety-related information was shared with all team members in the ward (P 25)
		(ii) It was a great help in knowing and communicating with the up-to-date protocols in a situation where the protocols related to COVID-19 were constantly changing (P 22)
		(iii) Huddles provided us an opportunity to gather together and communicate in a positive way (P 56)

Challenges for huddle participation (58)	Heavy workload (53)	(i) When I was too busy due to high workloads, it was difficult to participate huddles (P 11)
		(ii) Under the time pressure, it was a difficult for everyone to get together on time (P 52)
	Change in patients' condition (11)	(i) I was not able to participate in huddles when my patient's condition deteriorated and thus i had to urgently notify the doctor (P 4)
	Lack of awareness about huddles (7)	(i) It was difficult for everyone to participate due to persistent demands from patients or their families during huddles (P 55)
		(ii) Patients, physicians, and other professionals did not understand much about huddles, so huddles were often interrupted (P 69)

Recommendations for future huddles (7)	Recommendations for future huddles (7)	(i) Digital huddle board would be nicer than the white board (P 62)
		(ii) It seems that a plan should be prepared so that huddles can be operated effectively even when the unit manager is not present (P 68)
		(iii) Setting an alarm for a huddle would be helpful (P 75)

## Data Availability

The participants of this study did not give written consent for their data to be shared publicly, so due to the sensitive nature of the research, supporting data are not available.
